# Supplementary material for: Integrated virtual reality and musical biofeedback for intensity-guided training on stationary cycling: A comparative feasibility study
Source: PLOS Digit Health. 2026 Jul 22;5(7):e0001203. doi: 10.1371/journal.pdig.0001203 (PMC13390863; doi:10.1371/journal.pdig.0001203)
Supplement: S1 Table — Comprehensive performance measures during the first 9-minute exercise session (Set 1: 3 min baseline, 3 min + 15% intensity, 3 min baseline) for visual-only, musical-only, and combined audiovisual feedback conditions. Values are presented as median [IQR]. Metrics include: percentage of time spent within target speed zone (% time in zone), rate of exits from target zone (exits/min), median recovery time following zone exits (seconds), and number of sustained deviations (periods >5 consecutive seconds outside target zone). (PDF) [file pdig.0001203.s005.pdf]

| Metric | Visual | Musical | Combined |
| --- | --- | --- | --- |
| % Time in zone | 89.42 [80.41–95.88] | 88.90 [86.55–92.75] | 95.75 [92.10–97.60] |
| Exits/min | 1.48 [1.00–3.05] | 14.52 [11.19–23.07] | 1.79 [1.31–2.18] |
| Recovery time (s) | 2.48 [0.87–3.84] | 0.21 [0.19–0.25] | 1.06 [0.92–1.43] |
| Sustained deviations | 2.50 [1.00–4.00] | 0.50 [0.00–1.00] | 0.50 [0.00–3.00] |

S1 Table: values shown as Median [Q1–Q3]. See S2 Table for statistical comparisons.
