# Supplementary material for: Integrated virtual reality and musical biofeedback for intensity-guided training on stationary cycling: A comparative feasibility study
Source: PLOS Digit Health. 2026 Jul 22;5(7):e0001203. doi: 10.1371/journal.pdig.0001203 (PMC13390863; doi:10.1371/journal.pdig.0001203)
Supplement: S2 Table — Statistical comparisons across feedback modalities for performance measures during Set 1. Test selection was based on data distribution and variance homogeneity. Effect sizes (η2) are interpreted as small (≥0.01), medium (≥0.06), and large (≥0.14). Post-hoc pairwise comparisons were conducted with Bonferroni-adjusted significance threshold (α = 0.0167). (PDF) [file pdig.0001203.s006.pdf]

Metric	Test	p-value	η^2	Post-hoc (p-corrected)
% Time in zone	Welch ANOVA	0.0335*	0.155	—
Exits/min	Welch ANOVA	0.0006**	0.661	M-V (0.0005), M-C (0.0005)
Recovery time (s)	Welch ANOVA	<0.0001**	0.479	M-V (0.0022), M-C (0.0001)
Sustained deviations	Kruskal-Wallis	0.0422*	0.173	M-V (0.0109)

S2 Table. * $p < 0.05$, ** $p < 0.01$. Post-hoc: Games-Howell or Mann-Whitney ($\alpha = 0.0167$). V = Visual, M = Musical, C = Combined.
