# Supplementary material for: Integrated virtual reality and musical biofeedback for intensity-guided training on stationary cycling: A comparative feasibility study
Source: PLOS Digit Health. 2026 Jul 22;5(7):e0001203. doi: 10.1371/journal.pdig.0001203 (PMC13390863; doi:10.1371/journal.pdig.0001203)
Supplement: S3 Table — Comprehensive performance measures during the second 9-minute exercise session (Set 2: 3 min baseline, 3 min -15% intensity, 3 min baseline) for visual-only, musical-only, and combined audiovisual feedback conditions. Values are presented as median [IQR]. Metrics include: percentage of time spent within target speed zone (% time in zone), rate of exits from target zone (exits/min), median recovery time following zone exits (seconds), and number of sustained deviations (periods >5 consecutive seconds outside target zone). (PDF) [file pdig.0001203.s007.pdf]

Metric	Visual	Musical	Combined
% Time in zone	97.28 [87.79–99.81]	95.17 [86.26–95.99]	98.13 [97.10–99.48]
Exits/min	0.38 [0.11–1.84]	9.41 [7.19–21.67]	0.54 [0.32–0.55]
Recovery time (s)	1.60 [0.94–2.77]	0.22 [0.21–0.23]	1.80 [0.66–2.21]
Sustained deviations	1.00 [0.00–4.00]	0.00 [0.00–0.00]	0.50 [0.00–1.00]

S3 table: values shown as Median [Q1–Q3]. See S4 Table for statistical comparisons.
