# Supplementary material for: Integrated virtual reality and musical biofeedback for intensity-guided training on stationary cycling: A comparative feasibility study
Source: PLOS Digit Health. 2026 Jul 22;5(7):e0001203. doi: 10.1371/journal.pdig.0001203 (PMC13390863; doi:10.1371/journal.pdig.0001203)
Supplement: S4 Table — Statistical comparisons across feedback modalities for performance measures during Set 2. Test selection was based on data distribution and variance homogeneity. Effect sizes (η2) are interpreted as small (≥0.01), medium (≥0.06), and large (≥0.14). Post-hoc pairwise comparisons were conducted with Bonferroni-adjusted significance threshold (α = 0.0167). (PDF) [file pdig.0001203.s008.pdf]

| **Metric** | **Test** | **p-value** | $\eta^2$ | **Post-hoc (p-corrected)** |
|---|---|---|---|---|
| % Time in zone | Welch ANOVA | 0.0164* | 0.190 | M-C (0.0077) |
| Exits/min | Kruskal-Wallis | 0.0001** | 0.573 | M-V (0.0002), M-C (0.0002) |
| Recovery time (s) | Welch ANOVA | 0.0009** | 0.266 | M-C (0.0014) |
| Sustained deviations | Kruskal-Wallis | 0.0369* | 0.249 | — |

S4 Table. * $p < 0.05$, ** $p < 0.01$. Post-hoc: Games-Howell or Mann-Whitney ($\alpha = 0.0167$). V = Visual, M = Musical, C = Combined.
