# Supplementary material for: Integrated virtual reality and musical biofeedback for intensity-guided training on stationary cycling: A comparative feasibility study
Source: PLOS Digit Health. 2026 Jul 22;5(7):e0001203. doi: 10.1371/journal.pdig.0001203 (PMC13390863; doi:10.1371/journal.pdig.0001203)
Supplement: S5 Table — Weighted dimension scores (0–100% scale) for the six subscales of the NASA Task Load Index and the overall weighted workload score across visual-only, musical-only, and combined audiovisual feedback conditions. Values are presented as median [IQR]. Dimensions assessed include: Mental Demand (MD), Physical Demand (PD), Temporal Demand (TD), Performance (PE), Effort (EF), and Frustration (FR). Higher scores indicate greater workload for all dimensions except Performance, where higher scores indicate better perceived performance. (PDF) [file pdig.0001203.s009.pdf]

Dimension	Visual	Musical	Combined
MD	4.5 [1.3–11.0]	5.2 [2.7–6.7]	2.3 [0.7–4.0]
PD	11.0 [7.0–14.7]	10.0 [1.3–14.0]	18.7 [16.0–26.7]
TD	3.5 [1.0–10.0]	6.0 [0.7–12.0]	4.3 [3.3–10.0]
PE	10.3 [6.0–14.0]	20.5 [6.7–30.0]	9.7 [9.3–17.0]
EF	14.0 [5.0–23.3]	10.8 [6.7–12.0]	21.3 [18.3–23.3]
FR	0.0 [0.0–0.0]	0.2 [0.0–2.0]	0.0 [0.0–0.0]
TLX_{total}	54.8 [48.3–58.7]	51.2 [42.0–70.0]	64.5 [51.3–69.7]

S5 Table: values shown as Median [Q1–Q3]. See S6 Table for statistical comparisons. *Abbreviations:* MD, Mental Demand; PD, Physical Demand; TD, Temporal Demand; PE, Performance; EF, Effort; FR, Frustration.
