# Supplementary material for: Integrated virtual reality and musical biofeedback for intensity-guided training on stationary cycling: A comparative feasibility study
Source: PLOS Digit Health. 2026 Jul 22;5(7):e0001203. doi: 10.1371/journal.pdig.0001203 (PMC13390863; doi:10.1371/journal.pdig.0001203)
Supplement: S6 Table — Statistical comparisons across feedback modalities for cognitive workload dimensions. Test selection was based on data distribution and variance homogeneity. Effect sizes (η2) are interpreted as small (≥0.01), medium (≥0.06), and large (≥0.14). Post-hoc pairwise comparisons were conducted with Bonferroni-adjusted significance threshold (α = 0.0167). Combined feedback showed significantly higher physical demand compared to both musical and visual conditions, and significantly higher effort compared to visual-only feedback. (PDF) [file pdig.0001203.s010.pdf]

Dimension	Test	p-value	η^2	Post-hoc (p-corrected)
MD	Kruskal-Wallis	0.1486	0.108	—
PD	ANOVA	0.0016**	0.378	M-C (0.0090), V-C (0.0025)
TD	ANOVA	0.8039	0.016	—
PE	ANOVA	0.1164	0.147	—
EF	ANOVA	0.0189*	0.255	V-C (0.0147)
FR	Kruskal-Wallis	0.1725	0.098	—
$\text{TLX}_{\text{total}}$	ANOVA	0.2450	0.099	—

S6 Table. * $p < 0.05$, ** $p < 0.01$. Post-hoc: Tukey-Kramer or Mann-Whitney ($\alpha = 0.0167$). V = Visual, M = Musical, C = Combined. *Abbreviations:* MD, Mental Demand; PD, Physical Demand; TD, Temporal Demand; PE, Performance; EF, Effort; FR, Frustration.
