# Supplementary material for: Integrated virtual reality and musical biofeedback for intensity-guided training on stationary cycling: A comparative feasibility study
Source: PLOS Digit Health. 2026 Jul 22;5(7):e0001203. doi: 10.1371/journal.pdig.0001203 (PMC13390863; doi:10.1371/journal.pdig.0001203)
Supplement: S7 Table — Scores (1–5 Likert scale) from the e-Rubric usability questionnaire across visual-only, musical-only, and combined audiovisual feedback conditions. Values are presented as median [IQR]. Dimensions evaluated include: Usefulness, Ease of Use, Ease of Learning, Satisfaction and Overall Usability. All three feedback modalities achieved high usability scores across dimensions, with median scores generally exceeding 4.0 on the 5-point scale. (PDF) [file pdig.0001203.s011.pdf]

**Dimension**	**Visual**	**Musical**	**Combined**
Usefulness	4.25 [3.50–4.50]	4.50 [4.50–5.00]	4.50 [4.00–4.50]
Ease of Use	4.21 [4.00–4.43]	4.64 [4.43–4.86]	4.36 [4.14–4.71]
Ease of Learning	4.00 [3.25–4.25]	4.25 [3.25–4.75]	4.12 [3.50–4.25]
Satisfaction	4.38 [3.50–4.75]	4.75 [4.50–5.00]	4.25 [4.00–4.75]
Overall Usability	4.21 [3.76–4.38]	4.57 [4.29–4.62]	4.24 [3.95–4.62]

S7 Table: values shown as Median [Q1–Q3]. All scores rated on 1–5 Likert scale. See S8 Table for statistical comparisons.
