# Supplementary material for: Integrated virtual reality and musical biofeedback for intensity-guided training on stationary cycling: A comparative feasibility study
Source: PLOS Digit Health. 2026 Jul 22;5(7):e0001203. doi: 10.1371/journal.pdig.0001203 (PMC13390863; doi:10.1371/journal.pdig.0001203)
Supplement: S8 Table — Statistical comparisons across feedback modalities for user experience and usability dimensions assessed through the e-Rubric questionnaire. Test selection was based on data distribution and variance homogeneity. Effect sizes (η2) are interpreted as small (≥0.01), medium (≥0.06), and large (≥0.14). Post-hoc pairwise comparisons were conducted with Bonferroni-adjusted significance threshold (α = 0.0167). Although Satisfaction showed a significant global effect, no pairwise comparisons reached significance after correction. (PDF) [file pdig.0001203.s012.pdf]

Dimension	Test	p-value	η^2	Post-hoc (p-corrected)
Usefulness	Kruskal-Wallis	0.1400	0.123	—
Ease of Use	ANOVA	0.0995	0.157	—
Ease of Learning	ANOVA	0.8570	0.011	—
Satisfaction	Welch ANOVA	0.0418*	0.169	—
Overall Usability	ANOVA	0.0698	0.179	—

S8 Table. * $p < 0.05$. Post-hoc: Games-Howell ($\alpha = 0.0167$). V = Visual, M = Musical, C = Combined. Although Satisfaction showed a significant global effect ($p = 0.0418$), no pairwise comparisons reached significance after correction (M vs V: $p = 0.0583$, M vs C: $p = 0.0411$).
