# Supplementary material for: Integrated virtual reality and musical biofeedback for intensity-guided training on stationary cycling: A comparative feasibility study
Source: PLOS Digit Health. 2026 Jul 22;5(7):e0001203. doi: 10.1371/journal.pdig.0001203 (PMC13390863; doi:10.1371/journal.pdig.0001203)
Supplement: S1 Appendix — The questionnaire was used to evaluate usability and user experience. It was administered in Spanish, the participants’ native language. The English translations below are provided for reference. The questionnaire comprises 21 applicable items rated on a 5-point Likert scale, grouped into four dimensions following the USE framework: Usefulness, Ease of Use, Ease of Learning, and Satisfaction. Items originally worded negatively were reverse-scored during analysis so that higher scores consistently indicate better usability across all items. Notice that item 6 was removed since it was unrelated. (PDF) [file pdig.0001203.s013.pdf]

Item No.	Question (English translation)	Dimension
1	It was easy to use, without great effort.	Ease of Use
2	Incompatibilities appeared during use that made handling difficult.	Ease of Use
3	The environment was pleasant.	Satisfaction
4	The tool was exhausting and frustrating.	Ease of Use
5	It can be used without the need for prior explanations.	Ease of Learning
6	It was problematic to save and display your work.	Removed
7	It was motivating.	Satisfaction
8	I would prefer to have used another known tool instead of this one.	Satisfaction
9	It was simple, without unnecessary additions or complications.	Ease of Use
10	I felt insecure because the tool could be damaged by what I was doing.	Usefulness
11	I found the expected functions well integrated.	Satisfaction
12	The tool does not encourage its use.	Satisfaction
13	The tool can be understood very quickly by most users.	Ease of Learning
14	I encountered technical problems.	Usefulness
15	It performs the functions adequately to my expectations.	Usefulness
16	It takes more time with this tool than with another.	Usefulness
17	In case of need, I would use the tool again in the future.	Satisfaction
18	The language used was difficult to understand to interact with the tool.	Ease of Learning
19	It requires the help of a computer technician.	Ease of Use
20	I disagree with the graphical design of the tool.	Satisfaction
21	I would recommend this tool to other users.	Satisfaction
22	Overall, I am satisfied with the tool.	Satisfaction
